# Factor Analysis of the Brazilian Version of UPPS Impulsive Behavior Scale

**DOI:** 10.3389/fpsyg.2017.00622

**Published:** 2017-04-24

**Authors:** Cristina Y. N. Sediyama, Ricardo Moura, Marina S. Garcia, Antonio G. da Silva, Carolina Soraggi, Fernando S. Neves, Maicon R. Albuquerque, Setephen P. Whiteside, Leandro F. Malloy-Diniz

**Affiliations:** ^1^Faculty of Medicine, Federal University of Minas Gerais, Belo HorizonteBrazil; ^2^Department of Basic Psychological Processes, Institute of Psychology, University of Brasília, BrasíliaBrazil; ^3^Molecular Medicine Department at Federal University of Minas Gerais - INCT, Federal University of Minas Gerais, Belo HorizonteBrazil; ^4^Latin American Psychiatry Society, Porto University, PortoPortugal; ^5^Department of Psychology, Federal University of Minas Gerais, Belo HorizonteBrazil; ^6^Department of Sports, Federal University of Minas Gerais, Belo HorizonteBrazil; ^7^Mayo Clinic, Rochester, MNUSA; ^8^Brazilian Society of Dual Pathology, Ilumina Neurosciences and Mental Health, Belo HorizonteBrazil

**Keywords:** impulsivity, UPPS Impulsive Behavior Scale, psychometric, UPPS (urgency, lack of premeditation, lack of perseverance, sensation seeking), executive function

## Abstract

**Objective:** To examine the internal consistency and factor structure of the Brazilian adaptation of the UPPS Impulsive Behavior Scale.

**Methods:** UPPS is a self-report scale composed by 40 items assessing four factors of impulsivity: (a) urgency, (b) lack of premeditation; (c) lack of perseverance; (d) sensation seeking. In the present study 384 participants (278 women and 106 men), who were recruited from schools, universities, leisure centers and workplaces fulfilled the UPPS scale. An exploratory factor analysis was performed by using Varimax factor rotation and Kaiser Normalization, and we also conducted two confirmatory analyses to test the independency of the UPPS components found in previous analysis.

**Results:** Results showed a decrease in mean UPPS total scores with age and this analysis showed that the youngest participants (below 30 years) scored significantly higher than the other groups over 30 years. No difference in gender was found. Cronbach’s alpha, results indicated satisfactory values for all subscales, with similar high values for the subscales and confirmatory factor analysis indexes also indicated a poor model fit. The results of two exploratory factor analysis were satisfactory.

**Conclusion:** Our results showed that the Portuguese version has the same four-factor structure of the original and previous translations of the UPPS.

## Introduction

Impulsivity is an important aspect of personality and is a central role in many forms of psychopathology. In general, impulsivity has been broadly defined as quick unplanned actions that lead to thoughtless behaviors and a tendency to act with a lower level of planning compared to individuals of similar intellectual level (Moeller et al., 2001). The lack of consensus regarding the definition of impulsivity is probably one of the main reasons for the great variation of results among studies that assess impulsivity (Malloy-Diniz et al., 2009). Such inconsistencies hamper efforts to understand the role of impulsivity in many forms of psychopathology, such as, substance abuse (Verdejo-García et al., 2007) and bipolar disorder (Malloy-Diniz et al., 2011).

Impulsivity is present in several psychiatric conditions and predictor of severity of medical, employment, alcohol, drug, family/social, legal and psychiatric problems in individuals with substance dependence (Verdejo-García et al., 2007). For example, in patients with bipolar disorder, the manifestation of impulsivity has been linked to suicidal behavior, as well as activities with high output of negative consequences and low quality of life (Malloy-Diniz et al., 2009; Kim et al., 2013).

To validate the main existing models of impulsivity Whiteside and Lynam (2001) analyzed the responses of 437 undergraduates in 17 widely used measures of impulsivity. The study resulted in the four factors UPPS Impulsive Behavior Scale. The first factor is lack of premeditation, which is characterized by the inability to think about the consequences of a decision. The second factor relates to sensation seeking, which is the tendency of an individual to engage in exciting activities and the urge to live new experiences that may or may not be dangerous. The third factor is urgency that represents a tendency to act impulsively in the presence of negative emotions at the expense of long-term gains. The last factor is lack of perseveration, and is characterized by the difficulty of maintaining focus on a particular task.

The UPPS Impulsive Behavior Scale has been translated into many languages and has been found to have strong psychometric properties in French and German (i.e., internal consistency coefficients scale between 0.77 and 0.85) as well as exploratory and confirmatory factor analysis (CFA) replicating the original four factors (Van der Linden et al., 2006; Schmidt et al., 2008). The validation and structuring of the UPPS for the Brazilian student sample, will contribute for future analysis of researches of other cultures, and in the future it will assist in clinical profiling in Brazil.

For the reasons presented above, the current authors translated the UPPS to Portuguese (Sediyama et al., 2013). However, before the UPPS can be used in researches or clinical work in Brazil, the psychometric properties of the Portuguese UPPS have to be established. Therefore, the aim of the present study was to examine in a student sample the internal consistency and factor structure of Brazilian adaptation of the UPPS Impulsive Behavior Scale.

## Materials and Methods

### Participants

The study analyzed 384 participants (278 women and 106 men) that were convenient sample recruited from schools, universities, leisure centers, and workplaces. The mean age for female participants was 31-years-old (*SD* = 11.94) while male participants had a mean age of 34 years of age (*SD* = 13.89). Participants did not meet criteria for any psychiatric disorder relating to the MINI-Plus v5.0 (Amorim, 2000). Criterion analyses excluded participants younger than 18 or older than 62 years, with illiteracy, and with reported neurological disorders (such as cerebrovascular accident and epilepsy).

The participants were approached to in class, and asked to participate in the research by filling in the UPPS scale, socio-demographic scale, and MINI-Plus 5.0 (Amorim, 2000). Each candidate took an average of 60 min to finish.

The data from this study have all been drawn and have received ethical approval from the local research ethics committee of the Federal University of Minas Gerais, number ETIC 553/08. All participants provided written consent agreeing to the conditions before taking part of the experiment.

### Instruments

#### Portuguese Impulsive Behavior Scale (UPPS)

The original version (Whiteside and Lynam, 2001) was adapted to Portuguese by Sediyama (Sediyama et al., 2013). It is a self-report scale which consists of 45 items that address the four personality factors associated to impulsive behavior in a Likert-type format ranging from 1 to 4: (1) strongly agree, (2) partially agree; (3) partially disagree and (4) strongly disagree. Besides the total scores of impulsivity, the UPPS also provides the subscale scores of each subtype of impulsivity: lack of premeditation, urgency, sensation seeking and lack of perseverance.

#### Mini International Neuropsychiatric Interview (MINI Plus v.5)

The MINI Plus is a structured diagnostic interview compatible with DSM-III-R/IV and ICD-10. This instrument was developed for clinical practice and research in psychiatric and primary care settings. The MINI Plus is a more detailed version that mainly helps diagnose psychotic and mood disorders in DSM-IV (Amorim, 2000).

### Statistical Analysis

Descriptive statistics were calculated by mean and standard deviation. Normality of data distribution was verified by the Kolmogorov–Smirnov test. The internal consistency of the scale was calculated using the McDonald’s omega and Cronbach’s alpha. The One-way ANOVA with Tukey *post hoc* test were used to compare age groups. Kaiser-Meyer-Olkin (KMO) and Bartlett’s test of sphericity were used for the evaluation of model sufficiency (Field, 2009). High values of KMO (more than 0.70) and values lower than 0.05 of significance of Bartlett’s test probability indicate a satisfactory factor analysis (Field, 2009).

The analysis of the construct validity was done in two steps. First, a CFA was conducted to test the four-component structure of UPPS, with uncorrelated factors, found in previous studies (Magid and Colder, 2007). Second, an exploratory factor analysis (EFA) was performed. Weighted least squares method (WLSMV) estimator, once this estimator is recommended as a good alternative for items answered in an ordinal categorical scale (Chen et al., 2015). Geomin oblique rotation was used in EFA, and a range of indices were used to estimate how well the data fits the proposed model. These indexes included the chi-square and its corresponding *p-*value, the relative chi-square statistic, the root mean square error of approximation (RMSEA), the comparative fit index (CFI), and the Tucker-Lewis Index (TLI). Here we used widely adopted guidelines to interpret the adequacy of model fit, considering χ^2^ / *df* index less than 2, and RMSEA of 0.08 or lower, and both CFI and TLI with a value of 0.90 (Brown, 2006; Kline, 2011).

## Results

### Descriptive Statistics and Internal Consistency

A Kolmogorov–Smirnov test on the UPPS total scores indicated normal distribution of the data, *D* = 0.03, *p* > 0.05. In order to analyze the effect of age in UPPS scores the sample was divided in four age-groups: below 30, between 30 and 39, between 40 and 49, and above 50 years of live. Results showed a decrease in mean UPPS total scores with age, *F* = 7.37, *p* < 0.001. *Post hoc* analysis corrected for multiple comparisons showed that the youngest participants (below 30 years) scored significantly higher than the other groups (all *p*’s < 0.05), and all other comparisons did not reveal significant differences (all *p*’s > 0.05). Moreover, UPPS scores were also similar across gender, *t*(244) = 1.09, *p* > 0.05.

Answers to items 2, 3, 6, 7, 8, 10, 11, 14, 15, 18, 19, 21, 24, 25, 28, 29, 32, 33, 36, 37, 38, 21, 42, 44 and 45 (2 through 45), were reversed in order to keep the value of 1 corresponding to the lowest level of impulsivity, and the value of 4 to the highest level of impulsivity. Scores in the UPPS scale ranged from 49 to 139 points, with an average of 94.93 points (standard deviation = 15.39). **Table 1** shows means and standard deviation for each subscale of the UPPS.

**Table 1:** Cronbach’s alpha coefficient, mean, and standard deviation.

Scale	Mean score (*SD*)	Range	α (IC_95%_)
Lack of premeditation	20.32 (5.72)	11–44	0.87 (0.86–0.89)
Urgency	28.75 (7.40)	12–47	0.85 (0.83–0.87)
Sensation seeking	28.98 (8.47)	11–48	0.84 (0.82–0.87)
Lack of perseverance	19.20 (4.56)	10–32	0.75 (0.72–0.79)


Internal consistency of UPPS was assessed using McDonald’s omega and Cronbach’s alpha coefficient to measure scale reliability. For a structure with four factors, using maximum likelihood as the extraction method, the omega hierarchical (ω_h_) was 0.40, suggesting that around 40% of test variance is attributable to a general factor common to all items. The omega total (ω_t_), in turn, reached an index of 0.91, thus indicating that a large part of overall test variance (91%) is due to a general but also to the other four specific factors. Omega total (ω_t_) for the individual factors were also high (ω_t_ factor 1 = 0.91; ω_t_ factor 2 = 0.88; ω_t_ factor 3 = 0.83; ω_t_ factor 4 = 0.74). Additionally, the Cronbach’s alpha, results indicated satisfactory values for all subscales, with similar high values for the subscales; Lack of premeditation (0.87), Urgency (0.85), Sensation seeking (0.84), and a smaller value for the subscale Lack of perseverance (0.75). Item-total correlation (dropping corresponding items from total scores in order to avoid overestimated correlations) also corroborated these findings, as the coefficients were generally high (0.61 on average), and no item exhibited a coefficient less than 0.37 (**Table 1**).

### Construct Validity

The Kaiser-Meyer-Olkin (KMO) coefficient showed a sufficient magnitude (0.86), and the sphericity Bartlett’s test was significant (*p* < 0.001). First, we conducted a CFA on all items of the Brazilian version of UPPS. CFA evaluated the adequacy of fit of the orthogonal model four-factor solution. Results revealed a significant Chi-square statistic, χ^2^ = 2424.15, *p* < 0.05, *df* = 939, χ^2^ / *df* = 2/58. Other CFA indexes also indicated a poor model fit, CFI = 0.87, TLI = 0.87, and RMSEA = 0.065 (IC_95%_ = 0.062–0.068).

As CFA suggested that orthogonal model four-factor solution of the Brazilian version of UPPS was not suitable, the EFA was used to investigate the UPPS factorial structure.

As shown in **Table 2**, the initial EFA suggested that four-factor solution of the Brazilian version of UPPS was not suitable (see EFA1). Based on the Factor Loading, three items (20, 30, and 43) were excluded in the next analysis (EFA2) because showed large loading values in different factor than in the original theoretical structure.

**Table 2:** Fit indices for confirmatory factor analysis models.

	Number of items	Excluded items	χ^2^	gl	χ^2^/gl	CFI	TLI	RMSEA	Interpretation
AFE1	45	0	12820.84^∗^	990	12.95	0.94	0.93	0.048 (0.044–0.052)	Not suitable
AFE2	42	20, 30, 43	1341.29^∗^	699	1.92	0.94	0.93	0.050 (0.046–0.054)	Suitable


*^∗^*p* < 0.05.*

**FIGURE 1:**
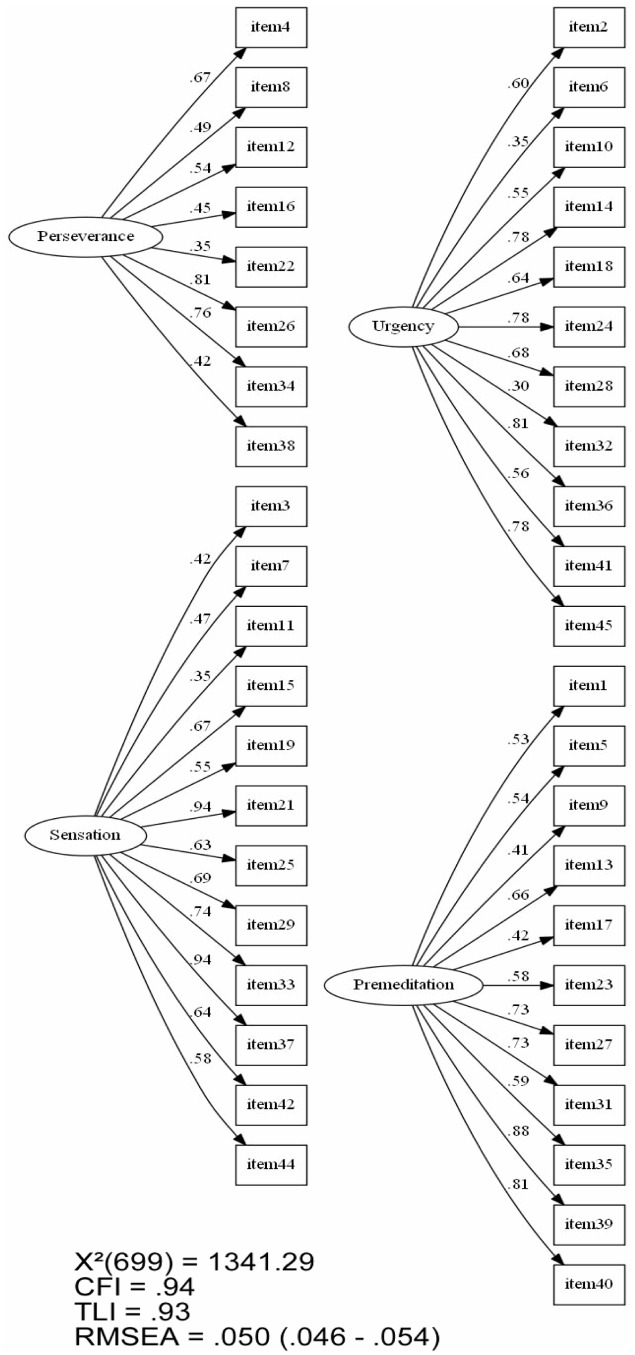
**Model fit indices for the final model by second exploratory factor analysis with factor loadings**.

The final version of four-factor solution of the Brazilian version of UPPS presented suitable (**Figure 1**) [χ^2^_(699)_ = 1341.29; *p* < 0.001; CFI = 0.94; TLI = 0.93; RMSEA = 0.050, with Geomin Rotated factor loadings greater or equal than 0.30 for all items].

Correlation between all four factors is present in **Table 3**. All latent variables but Sensation seeking correlated with at least one other variable. Similar findings were reported in previous studies (Van der Linden et al., 2006; Schmidt et al., 2008).

**Table 3:** Factor correlation.

Factor	Lack ofpremeditation	Urgency	Sensation seeking	Lack ofperseverance
Lack of premeditation	1			
Urgency	0.36	1		
Sensation seeking	0.11	0.09	1	
Lack of perseverance	0.48	0.17	0.08	1


## Discussion

The present study showed that results supported the four factors of the Portuguese version, applied in a student sample, and it was consistent with previous research, that found the same factors in UPPS Impulsive Behavior Scale (Whiteside and Lynam, 2001; Magid and Colder, 2007). In addition, the consistency of UPPS demonstrated in omega score, poor homogeneity in the scale and the Cronbach’s alpha, exploratory and CFA were satisfactory. These results are consistent with other translations that found internal consistency ranging from 0.75 to 0.87 (Van der Linden et al., 2006; Schmidt et al., 2008). Therefore, we can assume the four-dimensional structure and the distinct facets of Lack of premeditation, Urgency, Sensation seeking and Lack of perseverance as operationalized by the UPPS appear applicable to the Brazilian student population.

The adaptation of UPPS will provide an important tool for both clinical and research use. Since there’s no scale in Brazilian context to assess this factors of impulsive behavior. In addition, the identification of these four facets corroborates the literature that impulsivity is a heterogeneous category that includes several different features. However, more studies should be conducted to assess the psychometric properties of the scale.

Like previous research that demonstrate lifespan changes in impulsivity, this study found differences in UPPS according to age, (Steinberg et al., 2008; Burnett et al., 2012). In relation to sex differences, we did not observe this in our sample, but it should be emphasized that in a prior study reported such differences, using a different version of the UPPS, the UPPS-P (Cyders et al., 2007). This study reported that male participants differed from female ones in relation to positive urgency and sensation seeking (Cyders, 2013). Our hypothesis is that study sample was larger (*n* = 1,372 undergraduates), and probably favored a robust statistical analysis in relation to the sex difference. However, it should be noted that our study undergoes limitations, such as the vast majority of female samples, a problem also observed in the original study (Whiteside and Lynam, 2001) and others (Van der Linden et al., 2006; Schmidt et al., 2008).

The range application in other contexts, such as psychiatric Brazilian samples is necessary to show evidence of effectiveness studies which include the UPPS, and for example, whether these factors differ according to the psychiatric diagnosis or can be predictive of some disorder. In summary, our study showed that the Portuguese version of the UPPS has adequate psychometric properties, similar to those reported in different cultures.

## Author Contributions

All authors listed, have made substantial, direct and intellectual contribution to the work, and approved it for publication.

## Conflict of Interest Statement

The authors declare that the research was conducted in the absence of any commercial or financial relationships that could be construed as a potential conflict of interest.
